# Vitamin D Status and Vitamin D-Dependent Apoptosis in Obesity

**DOI:** 10.3390/nu12051392

**Published:** 2020-05-13

**Authors:** Igor N. Sergeev

**Affiliations:** Department of Health and Nutritional Sciences, South Dakota State University, Brookings, SD 57007, USA; igor.sergeev@sdstate.edu

**Keywords:** 1,25-dihydroxyvitamin D_3_, apoptosis, adipocytes, intracellular Ca^2+^, obesity, vitamin D status

## Abstract

The role of vitamin D in obesity appears to be linked to vitamin D insufficient/deficient status. However, mechanistic understanding of the role of vitamin D in obesity is lacking. We have shown earlier that the vitamin D hormonal form, 1,25-dihydroxyvitamin D_3_ (1,25(OH)_2_D_3_), induces cell death by apoptosis in mature adipocytes. This effect of the hormone is mediated by the cellular Ca^2+^ signaling pathway: a sustained increase of intracellular (cytosolic) Ca^2+^ concentration followed by activation of Ca^2+^-dependent initiators and effectors of apoptosis. In recent animal studies, we demonstrated that low vitamin D status is observed in diet-induced obesity (DIO). High intake of vitamin D_3_ in DIO decreased the weight of white adipose tissue and improved biomarkers related to adiposity and Ca^2+^ regulation. The anti-obesity effect of vitamin D (1,25(OH)_2_D_3_) in DIO was determined by the induction of Ca^2+^-mediated apoptosis in mature adipocytes executed by Ca^2+^-dependent apoptotic proteases (calpains and caspases). Thus, a high intake of vitamin D in obesity increases vitamin D nutritional status and normalizes vitamin D hormonal status that is accompanied by the reduction of adiposity. Overall, our findings imply that vitamin D may contribute to the prevention of obesity and obesity-related diseases and that the mechanism of the anti-obesity effect of 1,25(OH)_2_D_3_ includes induction of Ca^2+^-mediated apoptosis in adipocytes.

## 1. Introduction

Vitamin D is considered important for maintaining good health and preventing disease, however, the causality of those claims has not been mechanistically or probabilistically substantiated [1,2,3]. Vitamin D_3_ is the precursor of a secosteroid hormone 1,25-dihydroxyvitamin D_3_ (1,25(OH)_2_D_3_), which regulates a number of cellular functions, including the process of apoptotic cell death [4,5,6,7,8]. The mechanisms of hormonal effects of 1,25(OH)_2_D_3_ underlie its role in the regulation of cellular Ca^2+^ signaling, which, in turn, mediates a number of cellular responses, particularly Ca^2+^-mediated apoptosis [9,10,11,12].

Vitamin D deficiency and insufficiency (defined as a decreased concentration in blood of the transport form of this vitamin, 25-hydroxyvitamin D (25(OH)D)) appears to be associated with obesity [13]. The hormone 1,25(OH)_2_D_3_ plays a role in remodeling of adipose tissue via apoptotic Ca^2+^ signaling [14,15,16], and removal of mature adipocytes via 1,25(OH)_2_D_3_/Ca^2+^-mediated apoptosis may contribute to the maintenance of body weight and promotion of weight loss.

The purpose of this review is to discuss the potential link of vitamin D status to obesity and the role of 1,25(OH)_2_D_3_ in the regulation of adiposity, with emphasis on signaling pathways that involve vitamin D-dependent regulators, initiators, and effectors activated via 1,25(OH)_2_D_3_-induced cellular Ca^2+^ signaling. Understanding the role of vitamin D nutritional and hormonal status in obesity and mechanisms of 1,25(OH)_2_D_3_ in regulation of apoptosis in adipose tissue is important because it may influence the development of dietary recommendations for vitamin D and lead to the discovery of novel therapeutic and preventive modalities for this disease.

## 2. Vitamin D Status in Health and Obesity

Vitamin D status can be defined as sufficient, insufficient, or deficient. It depends on the level of exposure of unprotected skin to sun/UVB light and dietary/supplementary intake of this vitamin. The U.S. Institute of Medicine (IOM) [17,18], the Endocrine Society and the American Association of Clinical Endocrinologists [19], the European Food Safety Authority [20], and health regulatory agencies of several European countries [21,22] have established recommended vitamin D intake or defined vitamin D status (concentration of 25(OH)D in blood) as sufficient and deficient. Elevated and high vitamin D intake amounts (above the current upper-level intake of 4000 IU per day as defined by the IOM) have been and are currently promoted as beneficial for maintaining optimal health and preventing numerous diseases [3,19,23]. A mechanistic approach can provide needed clarity in defining vitamin D status, particularly the importance of measuring 25(OH)D concentration in blood as an indicator of vitamin D sufficiency. I would provocatively argue that vitamin D, as a hormonal precursor, requires such an approach for establishing its dietary requirements and defining its nutritional status or—a somewhat heretical view—it is not possible to establish dietary requirements for, and optimal status of, vitamin D (25(OH)D) based on currently used approaches.

The normal, physiological concentration of the active, hormonal form of vitamin D_3_, 1,25(OH)_2_D_3_, in blood and target tissues of healthy adults is homeostatically regulated and maintained regardless of circulating concentration of its precursor, 25(OH)D_3_ [4,5,21]. This implies that it is irrelevant (in healthy individuals under normal physiological and appropriate environmental conditions) how much of vitamin D_3_ or 25(OH)D_3_ is available for the biosynthesis of 1,25(OH)_2_D_3_ if it is sufficient/adequate for maintaining the circulating concentration of 1,25(OH)_2_D_3_ at the physiological (“normal”) level. The amount of available substrate, 25(OH)D_3_, is not a limiting factor for the production of 1,25(OH)_2_D_3_ under those conditions. For example, it would mean that intake/production of 600 IU vitamin D per day (the current recommendation), intake of 4000 IU (the current upper-level intake of vitamin D per day), the concentration of 25(OH)D in blood 20 ng/mL (low “normal” level) or 100 ng/mL (the highest level observed with excessive sun exposure) are all “sufficient” for maintaining the circulating concentration of 1,25(OH)_2_D_3_ at the physiological level. The concentration of 1,25(OH)_2_D_3_ in blood is three orders of magnitude lower than the “normal” level of 25(OH)D_3_ (in picomolar vs. nanomolar range) and the life of 1,25(OH)_2_D_3_ in circulation is shorter than that of 25(OH)D_3_ [4,5,21], which, again, clearly indicates a “sufficient” substrate–product relationship in this enzymatic reaction. High (yet not toxic) levels of vitamin D intake or prolonged sun/UVB exposure can significantly increase 25(OH)D concentration in blood, which increases vitamin D nutritional status/reserves so that physiological functions of vitamin D can be adequately performed for several months. Extra-renal production of 1,25(OH)_2_D_3_ utilizes 25(OH)D_3_ in situ, but it does not appear to contribute to maintaining the circulating 1,25(OH)_2_D_3_ level and the possible involvement of such production in paracrine/autocrine regulation of cellular functions is not clear [6,7].

An important point to note is that in the enzymatic reaction producing 1,25(OH)_2_D_3_, the substrate, 25(OH)D_3_, mechanistically functions not as a rate-limiting, but rather as an inhibitory substrate (i.e., high concentrations of 25(OH)D_3_ in the blood will inhibit production of 1,25(OH)_2_D_3_ in kidneys); moreover, the metabolism of 25(OH)D_3_ shifts toward 24,25-dihydroxyvitamin D_3_ at high concentrations of this substrate [4,21,24,25].

Thus, in the context discussed, it can be argued that establishing the optimal levels of vitamin D intake and the “normal” values of 25(OH)D concentration in the blood are not particularly relevant from a mechanistic perspective.

Biological responses to 1,25(OH)_2_D_3_ are determined by the concentration, spatial and temporal distribution, and the ligand- and DNA-binding activities of vitamin D receptors (VDRs) in the cell. VDRs at approximately 50% occupation level by its ligand (1,25(OH)_2_D_3_) mediate maximum genomic and non-genomic cellular responses, and this binding level is achieved at the physiological (picomolar) concentrations of 1,25(OH)_2_D_3_ [1,2,26,27]. The mechanistic conclusion is that the circulating concentration of the hormone 1,25(OH)_2_D_3_ must be maintained at the precise, narrow-range level within a broad range of concentrations of 25(OH)D_3_, thus permitting 1,25(OH)_2_D_3_ to mediate, via VDRs, cellular responses and perform normally, in a highly adaptive and homeostatic fashion its physiological functions even at the “insufficient” vitamin D status (similar to the functioning of other steroid hormones).

It is also worth mentioning that that rickets and osteomalacia are observed when 25(OH)D is “undetectable” in blood (below 0.5–5 ng/mL depending on the assay) and that major physiological functions of 1,25(OH)_2_D_3_ (intestinal Ca^2+^ absorption and bone growth, mineralization, and remodeling) can be maintained at optimal level in healthy individuals at concentrations of 25(OH)D in the range of 10–20 ng/mL (probably, even lower levels can be sufficient because they will not limit the production of 1,25(OH)_2_D_3_). In this context, claims regarding widespread vitamin D “deficiency” and the benefits of measuring 25(OH)D routinely and/or in various populations should be considered with skepticism. The definition of vitamin D insufficiency as a 25(OH)D concentration in the range of 20–30 ng/mL [3,23] is not particularly relevant from the mechanistic and, probably, nutritional/health points of view.

The important rationale for increasing dietary vitamin D intake when skin exposure to sunlight is low (e.g., due to geographic location, time indoors, skin color, sunscreen use, etc.) and maintaining 25(OH)D concentration in the range of 30–60 ng/mL is the potential role of vitamin D in preventing certain diseases [1,2,3,23], but the mechanisms of those effects and their association with vitamin D status remain largely speculative. It is also important to emphasize that a sustained 25(OH)D concentration above 100–150 ng/mL can increase the risk of vitamin D toxicity. In the case of therapeutic effects of vitamin D, pharmacological concentrations of the hormone, 1,25(OH)_2_D_3_, in blood and target tissues are required [8,12,26,27], and those levels cannot be achieved with vitamin D supplementation or sunlight/UVB exposure due to the homeostatic regulation of 1,25(OH)_2_D_3_ production. Moreover, 1,25(OH)_2_D_3_ in pharmacological concentrations causes significant side effects (hypercalcemia, bone resorption, etc.), and evaluations (including clinical trials) of noncalcemic analogs of 1,25(OH)_2_D_3_ provided mixed or discouraging results [8,12].

The vitamin D-dependent mechanisms in disease and the causal relationship between vitamin D status and disease (except vitamin D-deficient rickets/osteomalacia and inherited vitamin D receptor/vitamin D metabolism disorders) have yet to be identified, although an increased risk of some diseases (including obesity) has been linked to vitamin D deficiency and insufficiency [1,2,6,13]. Concurrently, disease states can alter vitamin D functioning, e.g., obesity may lower vitamin D status due to the accumulation and immobilization of vitamin D metabolites in adipose tissue and impaired conversion of vitamin D compounds in the liver and kidneys as well as affect the activity of vitamin D receptors [6,13].

The risk of acute and chronic vitamin D toxicity should not be underestimated. Examples of acute nutritional toxicity/death include consumption of vitamin D_3_ concentrate in vegetable oil (used on poultry farms) as cooking oil 25OHD concentration in blood of these people was 200–1000 ng/mL) and consumption of polar bear liver (vitamin D and A toxicity) [4,21]. Examples of chronic toxicity include calcification of soft tissues, particularly the aorta and kidneys [4,21]. To avoid toxicity, intake at levels of more than 4000 IU per day (the official IOM upper-level intake recommendation) or 5000–10,000 IU per day (by Rx) for prolonged periods of time (over several months) should be avoided and not recommended (due to increased risk of kidney stones, soft tissues (particularly, vascular) calcification, cardiovascular disease, coronary artery disease, etc.). In pediatric patients (recommendation of the American Academy of Pediatrics), the maximum dose of vitamin D is 50,000 IU one time per week, not for more than two months. Interestingly, even larger doses of vitamin D have been used for prophylaxis of rickets in certain populations in several European countries (e.g., a single dose of 300,000 IU) with no apparent side effects. However, it needs to be considered that infants and very old individuals do not have fully expressed/functional vitamin D hydroxylases and vitamin D receptors as well as a fully developed/expressed system for intestinal Ca^2+^ absorption (calbindin-D), therefore, a large vitamin D intake can be irrelevant in those cases. Furthermore, it is worth noting that the 25(OH)D concentration in human breast milk (including mothers with “normal” 25(OH)D concentration in their blood) is “very low” (it is not sufficient to meet the Adequate Intake (AI) of breastfed infants as defined by the IOM), possibly indicating low requirements for vitamin D in, or inability to utilize vitamin D by, infants. The skin of most terrestrial mammals is well protected from sun exposure, but they are not vitamin D deficient in the wild, even at high latitudes (and they do not get much vitamin D by licking their fur). Naturally vitamin D-deficient naked mole rats have undetectable levels of 25(OH)D [13] but are perfectly healthy and only express a unique-for-mammals social behavior (resembling that of social insects), extreme longevity (lifespan over 30 years), and are “naked” (either because of vitamin D deficiency or living underground). On the other hand, there is an interesting hypothesis that a mutation resulting in the light skin facilitated the migration of humans out of the tropics by allowing sufficient vitamin D production [13].

The controversial points made above emphasize that, in discussing vitamin D status in health and disease, it is critical to consider that the normal, physiological concentration of the active, hormonal form of vitamin D_3_—1,25(OH)_2_D_3_—in the blood and target tissues of healthy adults is regulated and maintained within a broad range of concentrations of its precursor, 25(OH)D_3_. I do not claim that sufficient (yet to be appropriately defined as such) vitamin D status is not critically important for maintaining good health and preventing diseases but suggest that increasing vitamin D intake above the levels currently recommended by the IOM, probably, will not provide additional health benefits. Conversely, vitamin D status (25(OH)D concentration in blood) can serve as an excellent marker of good health [6], so that many of the variables that contribute to decreased 25(OH)D concentration (unhealthy dietary patterns, low level of physical activity, aging, dark skin) are also risk factors for the development of diseases, including obesity. Therefore, mechanistic studies and clinical intervention trails are critical for resolving the uncertainty regarding potential benefits of increasing vitamin D intake for maintaining good health and preventing disease.

## 3. Vitamin D-Mediated Apoptosis and Obesity

Apoptosis, a highly regulated form of cell death, is the main mechanism for controlling cell numbers in most tissues [11,28,29,30,31,32,33,34,35,36,37]. The remodeling of adipose tissue via apoptosis is critical for maintaining an appropriate number of adipocytes by eliminating mature, oversized cells and, thus, helping to sustain the normal adipose tissue and body mass. Obesity due to increased adipose tissue mass can result from an increase in adipocyte number (hyperplasia) and/or an increase in adipocyte size (hypertrophy) [13]. An adequate rate of adipocyte apoptosis may prevent the excessive accumulation of adipose tissue, whereas an increased rate of apoptosis will result in the loss of adipose tissue mass over time. It was established recently [13] that mature, differentiated adipocytes undergo apoptosis and are not as stable a cell type as previously thought. Thus, the induction of adipocyte apoptosis can be employed as a strategy for the prevention and treatment of obesity because removal of adipocytes via this mechanism will result in a reduction in body fat and a long-lasting maintenance of weight loss.

The hypothesis that vitamin D can be a determinant of obesity risk is plausible from a mechanistic perspective because 1,25(OH)_2_D_3_ regulates the fate of adipocytes via apoptosis [1,2,6,10,14,37]. The apoptotic effect of vitamin D in these cells is mediated by nuclear VDRs and the regulatory effects of 1,25(OH)_2_D_3_ in Ca^2+^ signaling (through voltage-insensitive Ca^2+^ channels linked to membrane VDRs) [9,14,16]. The possible in situ production of 1,25(OH)_2_D_3_ in adipocytes and other cells in adipose tissue (e.g., macrophages) should be also considered.

Cellular Ca^2+^ signaling in adipocytes is critical among the signaling pathways linked to obesity [7,13,14], and this signaling is also a main target of the Ca^2+^ regulatory hormone 1,25(OH)_2_D_3_ [1,2,4]. Intracellular Ca^2+^ signals can trigger apoptosis in various cell types, but Ca^2+^-dependent mediators involved in the downstream apoptotic signaling have not been conclusively identified. We [7,8,9,10,12,29,30,32,33,34,35,36,37] and others [11,31] have shown that an increase in concentration of intracellular (cytosolic) Ca^2+^ ([Ca^2+^]_i_) occurs in the early and late stages of apoptosis. The critical characteristic of the apoptotic Ca^2+^ signal is a sustained, prolong, globalized, and nonoscillatory increase in [Ca^2+^]_i_, reaching elevated, but not cytotoxic, levels. The mechanism of action of intracellular Ca^2+^ in apoptotic pathways involves interactions of the cellular Ca^2+^ signals with Ca^2+^-dependent molecular targets in cells undergoing apoptosis. The Ca^2+^-dependent calpains and caspases appear to be the primary Ca^2+^-activated apoptotic effectors [7,8,14,29].

Specifically, we have shown [1,2,8,9,10] that a sustained increase in [Ca^2+^]_i_ rapidly signals the cell to enter the apoptotic pathway. The activation of Ca^2+^-dependent protease µ-calpain followed by the activation of Ca^2+^/calpain-dependent caspase-12 and other effector caspases (e.g., caspase-3) is responsible for the execution of Ca^2+^-mediated apoptosis. A lack of expression or low levels of cytosolic Ca^2+^ binding proteins (e.g., vitamin D-dependent calbindins) decrease Ca^2+^-buffering capacity of the cell, resulting in [Ca^2+^]_i_ increase and the induction of apoptosis [32,33]. We have further shown that 1,25(OH)_2_D_3_ differentially activates the voltage-dependent and voltage-insensitive Ca^2+^ entry pathways and differentially triggers Ca^2+^ release from the endoplasmic reticulum (ER) stores through the inositol 1,4,5-trisphosphate receptor/Ca^2+^ release channels (IP_3_Rs) and ryanodine receptor/Ca^2+^ release channels (RYRs) in several cell types [9,10,12,14,28]. It appears that Ca^2+^ signals triggered by 1,25(OH)_2_D_3_ in different cell types (e.g., transient vs. prolonged or oscillatory vs. sustained, steady Ca^2+^ fluxes) can be linked to both membrane and nuclear VDRs [8,9,10]. Remarkably, mature adipocytes demonstrate some of the Ca^2+^ handling characteristics (the lack of voltage-dependent Ca^2+^ channels, expression of voltage-insensitive Ca^2+^ channels, low cytosolic Ca^2+^ buffering capacity) conducive to the induction of Ca^2+^-mediated apoptosis with 1,25(OH)_2_D_3_ [14,15,16,33].

We have shown that 1,25(OH)_2_D_3_ induces apoptosis in adipocytes via a mechanism linked to the activation of Ca^2+^-dependent µ-calpain and Ca^2+^/calpain-dependent caspase-12 [14,15,16]. The treatment of mature adipocytes with 1,25(OH)_2_D_3_ induced, in a concentration- and time-dependent manner, a sustained increase in [Ca^2+^]_i_, and this increase led to the execution of apoptosis via activation of µ-calpain and caspase-12. The susceptibility of mature adipocytes to the Ca^2+^-elevating effect of 1,25(OH)_2_D_3_ appears linked to the low Ca^2+^-buffering capacity of these cells. These findings demonstrate that Ca^2+^-mediated apoptosis can be induced in mature adipocytes and that the apoptotic molecular targets activated by 1,25(OH)_2_D_3_ in these cells are Ca^2+^-dependent µ-calpain and caspase-12.

## 4. Vitamin D and Diet-Induced Obesity

A high-fat-diet-induced obesity (DIO) mouse model is characterized by obese phenotype, increased blood glucose concentration, and development of adiposity [6,13]. We have shown that DIO mice fed a diet with high vitamin D_3_ content demonstrate a decreased weight of adipose tissue and improved biomarkers of adiposity and vitamin D status [38,39]. The glucose and insulin concentrations in the blood of those mice were significantly decreased (to the levels measured in the non-obese control), whereas the concentration of adiponectin (an insulin-sensitizing adipokine) was increased. Moreover, low vitamin D status (a decreased plasma concentration of 25(OH)D_3_) and a decreased concentration of the hormone 1,25(OH)_2_D_3_ were observed in DIO mice. High vitamin D_3_ intake was accompanied by a significant increase in plasma concentration of 25(OH)D_3_ and 1,25(OH)_2_D_3_ to the levels corresponding to high vitamin D nutritional status and normal vitamin D hormonal status. High vitamin D_3_ intake was also associated with the induction of apoptosis (measured by morphological criteria, oligonucleosomal DNA fragmentation and ssDNA breaks) and the activation of Ca^2+^-dependent apoptotic proteases (calpain and caspase-12) in adipose tissue of DIO mice. Additionally, high vitamin D_3_ intake increased mineral (Ca and P) content in the bone of DIO mice via regulatory effects mediated by the 1,25(OH)_2_D_3_‒parathyroid hormone (PTH) axis (an increase in 1,25(OH)_2_D_3_ and Ca^2+^ concentration and a decrease in PTH concentration in blood) [39]. These results demonstrate that high vitamin D intake can effectively normalize biomarkers related to obesity and that the hormonal mechanism of these effects involves 1,25(OH)_2_D_3_. The findings also imply that increased vitamin D intake may contribute to the prevention of obesity and obesity-associated bone disorders.

## 5. Summary

**Figure 1:**
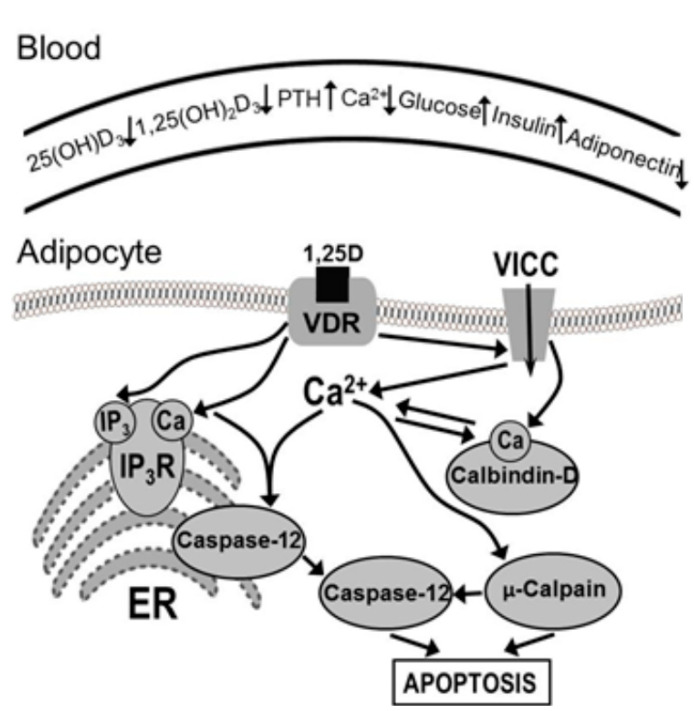
Mechanisms of regulation of intracellular Ca^2+^ and apoptosis by 1,25-dihydroxyvitamin D_3_ (1,25(OH)_2_D_3_) in obesity. VDR—vitamin D receptor; VICC—voltage-insensitive Ca^2+^ channels; IP_3_R—inositol 1,4,5-trisphosphate receptor/Ca^2+^ release channel; ER—endoplasmic reticulum; PTH—parathyroid hormone. Adapted from reference [2] with permission from Elsevier.

The studies reviewed here identified the novel 1,25(OH)_2_D_3_-regulated apoptotic pathway in adipocytes, namely: sustained increase in intracellular Ca^2+^ → activation of Ca^2+^-dependent calpain → activation of Ca^2+^/calpain-dependent caspase-12 → execution of apoptosis. These studies demonstrated that the 1,25(OH)_2_D_3_-activated molecular targets executing apoptosis in adipocytes are Ca^2+^-dependent calpain and Ca^2+^/calpain-dependent caspase-12 (Figure 1). Low vitamin D status in obesity and the role of 1,25(OH)_2_D_3_ in controlling adipose tissue mass in vivo by regulating adipocyte apoptosis may imply a mechanistic role for vitamin D in adiposity. Preclinical studies and clinical trials will be necessary to confirm the validity of 1,25(OH)_2_D_3_- and Ca^2+^-dependent molecular targets in apoptotic pathways for the prevention and treatment of obesity [40].

The cartoon provides a schematic representation of the possible mechanism of action of 1,25(OH)_2_D_3_ in inducing cellular Ca^2+^ signals and Ca^2+^-mediated apoptosis in mature adipocytes. Changes in the vitamin D status, Ca^2+^ regulatory hormones, and obesity-related biomarkers in the blood are also shown. This representation is largely based on the author’s studies employing an in vitro model of mature adipocytes and a mouse model of diet-induced obesity (DIO). Briefly, 1,25(OH)_2_D_3_ regulates Ca^2+^ entry from the extracellular space, Ca^2+^ mobilization from the intracellular stores, and intracellular (cytosolic) Ca^2+^ buffering. Moreover, 1,25(OH)_2_D_3_ induces Ca^2+^ influx from the extracellular space and Ca^2+^ mobilization from the endoplasmic reticulum (ER) stores via the high permeability voltage-insensitive Ca^2+^ channels (VICC) and IP_3_ receptor/Ca^2+^ release channel (IP_3_R), respectively. Vitamin D receptors (VDRs) are expressed in adipocytes, and they can be found associated with the cell membrane as well as in the nuclear and cytosolic compartments. Vitamin D-dependent Ca^2+^ buffering calbindin-D_9k_ appears to be expressed at a low level in mature adipocytes, which contributes to a decreased Ca^2+^ buffering capacity of these cells. 1,25(OH)_2_D_3_ activates the calpain/caspase-12-dependent apoptotic pathway in adipocytes by inducing the apoptotic Ca^2+^ signal—a sustained, prolong, globalized, and nonoscillatory increase in [Ca^2+^]_i_ reaching elevating (200–500 nM), but not cytotoxic (>1 μM) levels—via Ca^2+^ release from the ER stores and Ca^2+^ entry through the VICC. μ-Calpain activation by the sustained cytosolic Ca^2+^ signal is followed by activation (i.e., Ca^2+^-dependent translocation from the ER membrane to cytosol and calpain-dependent processing/cleavage) of Ca^2+^/calpain-dependent caspase-12. Activation of these proteases appears to be sufficient for the execution of apoptosis as evaluated by the hallmarks of this process: apoptotic changes of the plasma membrane (phosphatidylserine translocation from the inner to outer surface of the plasma membrane) and nuclear condensation and DNA fragmentation (oligonucleosomal fragments and ssDNA breaks).
